# Learning a sequence of motor responses to attain reward: a speed-accuracy trade-off

**DOI:** 10.1186/1471-2202-14-S1-P143

**Published:** 2013-07-08

**Authors:** Ignasi Cos, Pavel Rueda-Orozco, David Robbe, Benoît Girard

**Affiliations:** 1 ISIR, Université Pierre et Marie Curie, Paris, 75005, France; 2 UMR-7222, CNRS, Paris, 75005, France; 3 Institut de Neurobiologie de la Méditerranée (INMED), INSERM, Marseille, 13273, France

## 

The study of decision-making between goal directed actions with rodents has been often based on experimental tasks in which animals were trained to perform specific sequences of actions, such as lever presses or nose pokes [4], to attain reward. This supported the hypothesis of reinforcement learning as the underlying mechanism to acquire those behavioural sequences, putatively implemented by the basal-ganglia circuitry [1,3].

However, experimental evidence suggests that whenever we extend the complexity of the motor responses towards timely constrained behaviour, it starts reflecting an influence of costs related not only to reward, but rather a compromise between the motor factors relevant to the task, and the timely requirements to attain the goal [6]. To investigate this further, we took advantage of new behavioral protocol in which rats running on a treadmill need to estimate a fixed-temporal interval to obtain a reward [5]. Interestingly rats became proficients in this task by developping very stereotyped running trajectories. The establishment of these precise running kinematics occured progressively in a trial-and-error process that lasted between 2 to 3 months. At this point if we shortened the treadmill length, animals persisted in reproducing the previously learned kinematics even if doing so they stopped receiving reward. This is consistent with that these stereotyped running kinematics are motor habit [8].

To provide a theoretical backend for these results, we developed a model-free reinforcement learning model [7]. We excluded model-based algorithms because of the inability of the rats to exploit the previously learned behavior to accelerate their learning rate when the task changes. The specificity of this model is to count reward delivery as positive reward, but also efforts generated at each time step as negative rewards. The problem is thus a speed-accuracy trade-off process: the goal of the model is to generate the motor sequence that optimizes the ratio discounted reward/effort. The main result shows that, as long as the local time and speed are included into the characterization of the kinematic state, the model can replicate the same motor sequences. This suggests that these two pieces information are required to learn time-constrained motor sequences, and predicts that if a brain structure indeed learns these habitual sequences as the model does (our suggestion would be the sensorimotor circuits of the basal ganglia [2]), it should exhibit correlates with the same variables during the entire sequence.
